# Natal origin and migration pathways of Mekong catfish (*Pangasius krempfi*) using strontium isotopes and trace element concentrations in environmental water and otoliths

**DOI:** 10.1371/journal.pone.0252769

**Published:** 2021-06-10

**Authors:** Ngan Trong Tran, Maylis Labonne, Ming-Tsung Chung, Chia-Hui Wang, Kuo-Fang Huang, Jean-Dominique Durand, Chaiwut Grudpan, Bunyeth Chan, Huy Duc Hoang, Jacques Panfili

**Affiliations:** 1 Department of Ecology and Evolutionary Biology, University of Science, Ho Chi Minh City, Vietnam; 2 Vietnam National University, Ho Chi Minh City, Vietnam; 3 IRD, MARBEC (Univ Montpellier, Ifremer, CNRS, IRD), Montpellier, France; 4 The University of Tokyo, Atmosphere and Ocean Research Institute, Tokyo, Japan; 5 Department of Environmental Biology and Fisheries Science, National Taiwan Ocean University, Keelung, Taiwan; 6 Institute of Earth Sciences, Academia Sinica, Taipei, Taiwan; 7 Department of Fishery, Faculty of Agriculture, Ubon Ratchathani University, Ubon Ratchathani, Thailand; 8 Wonders of the Mekong Project, Inland Fisheries Research and Development Institute, Fisheries Administration, Phnom Penh, Cambodia; Tanzania Fisheries Research Institute, UNITED REPUBLIC OF TANZANIA

## Abstract

To improve our knowledge of the migration pathway of a highly threatened fish species along the Mekong River, strontium isotope ratios (^87^Sr/^86^Sr) and 18 trace element concentrations were measured in the water and in the otoliths of an anadromous catfish, *Pangasius krempfi*, to infer its natal origin and potential migration pathways. Water was sampled at 18 locations along the mainstream, tributaries and distributaries of the Mekong River. To check for accuracy and precision, measurements of the ^87^Sr/^86^Sr ratios and trace element concentrations were then compared in two laboratories that use different analytical methods. Differences in trace element concentrations between locations were not significant and could not, therefore, be used to discriminate between migration pathways. However, the Mekong mainstream, tributaries and distributaries could all be discriminated using Sr isotopes. The ^87^Sr/^86^Sr profiles recorded in *P*. *krempfi* otoliths showed that there were three contingents with obligate freshwater hatching and variable spawning sites along the Mekong mainstream, from Phnom Penh (Cambodia) to Nong Khai (Thailand) or further. After hatching, the fish migrated more or less rapidly to the Mekong Delta and then settled for most of their lifetime in brackish water. Spawning habitats and migration routes may be threatened by habitat shifts and the increasing number of hydropower dams along the river, especially the contingents born above Khone Falls (Laos). The conservation of *P*. *krempfi*, as well as other migratory fish in the Mekong River, requires agreements, common actions and management by all countries along the Mekong River. This study highlighted the importance of using both Sr/Ca and ^87^Sr/^86^Sr ratios to understand life history of anadromous fishes as the ^87^Sr/^86^Sr ratio in the water was shown to be less effective than the Sr/Ca ratio in identifying movements between different saline areas.

## Introduction

The Mekong River is the main river in South East Asia, it rises in the Tibetan Plateau, crosses six countries (China, Myanmar, Laos, Thailand, Cambodia, and Vietnam), and creates a delta before flowing out to sea. With a mainstream around 4,400 km long and a catchment area of 795,000 km² [1, 2], the Mekong River has the second highest inland fish biodiversity in the world, with more than 1,100 species [3, 4]. Wild fish are an important source of income and consumption for local populations [5]. Of all the major river systems in the world, the Mekong River and its Delta are probably the most affected by global climate change and human activities [6, 7]. Pollution and saline intrusions with rising sea levels due to climate change currently threaten the Delta [8–10], while the whole Mekong River is even more threatened by many dam-building projects that could have strong impacts, especially on fisheries [6]. With the dams currently under construction or planned in the near future on the Mekong mainstream and its tributaries, scientists are considering measures to mitigate the anthropogenic influences on all surrounding socio-ecosystems [6]. The numerous dams being built on the Mekong mainstream will have an negative impact on the migratory behavior of many fish species and on associated fisheries [6, 11]. By changing hydrological flow and habitat connectivity, the presence of dams impacts fish populations, by blocking possible migration pathways and reducing population size and potentially their genetic diversity [6, 12, 13]. Understanding the life history of migratory fish, as well as their key habitats in the Mekong River, has become essential for monitoring and managing fish communities and populations under threat.

It has been estimated that 87% of known fish species in the Mekong River are migratory (anadromous, catadromous, or potamodromous) [14] and that 50% of the catches include long-distance migrants [4]. The migration pathways in the Mekong River have been described based on the hydrological and morphological characteristics of the mainstream and on the feeding behavior of fish in different habitats [14]. In many species, these migration patterns are interconnected or overlapping. Among these patterns, the main migration route includes the mainstream from the Mekong Delta to the Khone Falls at the border between Cambodia and Laos, where annual floods affect large areas, and are essential for fishery productivity [14]. Nevertheless, our understanding of their migratory behavior is speculative, and the location of the spawning grounds and homing cues remain largely unknown.

Fish otoliths (ear stones) are biogenic carbonate structures deposited on a protein matrix, which grow throughout the fish’s lifetime with both daily and seasonal growth increments [15]. The otoliths are composed of 99% calcium carbonate, but, during formation, they incorporate trace elements from the water that can provide a detailed history of an individual fish’s environment and life events [16, 17]. The incorporation of elements is a complex process involving different physiological barriers. Elements are absorbed from the water through the gills or the intestine into the plasma and the endolymph surrounding the otolith, where they crystallize in the matrix [17]. The process is affected by various environmental and physiological factors, including salinity, temperature, ontogeny, metamorphosis, sex, growth, and diet [18–24]. Incorporation also depends on the element concerned, and there are many differences between them. For example, carbon isotopes and magnesium are related to physiological factors, whereas physiology is weakly related to strontium and lithium, and oxygen isotopes, barium, and manganese are related to past environments [24–26].

The study of fish environmental conditions and physiology is thus made possible by otolith chemistry. Strontium is known as the best proxy for ambient water salinity [20, 27], the Sr/Ca is the ideal variable to estimate fish movements between marine, brackish and fresh waters [28, 29], whereas it is not affected by water temperature [30]. When compared with water concentrations and age estimates, otolith strontium isotope ratios (^87^Sr/^86^Sr) can provide valuable information about the migration pathways of species that pass through different habitats [17, 31, 32]. More specifically, the strontium isotopic ratio (^87^Sr/^86^Sr) signatures in the water [33] and the otoliths provide evidence for the origin of individual migratory and non-migratory fish [34–39]. Duponchelle et al. [40] recently investigated the natal origin of an Amazonian giant catfish (*Brachyplatystoma rousseauxii*) and by comparing the Sr isotope ratios in otoliths and in the water over a very large spatial scale, these authors identified one of the longest fish migrations in freshwater: more than 4,000 km. However, the Sr isotope ratio is a proxy of natal origin in freshwater [40], and its use in saline areas is limited because the variation of ^87^Sr/^86^Sr value is less sensitive to changes in salinity when salinity is higher than 20 [29, 30].

The catfish *Pangasius krempfi* (Fang and Chaux 1949; Siluriformes, Pangasiidae) is one of the most important anadromous fishes in the Mekong River, and is classified as vulnerable in the International Union for Conservation of Nature (IUCN) Red List [41]. This species is widely distributed in the Mekong basin from China to Vietnam, but is primarily found in Laos, Cambodia, Thailand and Vietnam in both brackish waters and freshwaters [42, 43]. This catfish is one of the target species for fishery and aquaculture in the countries along the Mekong where it has high economic value in the local markets [43–46]. Although *P*. *krempfi* represents only around 2% of the total catches by Mekong fisheries, its economic value on landing is three times higher than that of more common species [45]. The domestication of *P*. *krempfi* has been investigated to improve catfish aquaculture in the Mekong Delta [46]. Information on the life history traits of the species is scarce in the literature. *P*. *krempfi* is believed to undertake a long migration from its growing areas in brackish waters in the Mekong delta over the rapids and deep pools of the Khone Falls in Laos for reproduction [44, 47] at the beginning of the rainy season [43]. However, the spawning grounds of *P*. *krempfi* and age at first sexual maturity and reproduction are not yet fully known [41, 43]. After hatching at the Khone Falls, the larvae are assumed to rapidly drift downstream, and juveniles and adults spend almost all their life in the Mekong Delta before migrating back upstream to spawn [14, 44, 47]. Living in the brackish Mekong Delta, this anadromous species is presumed to be sensitive to the construction of dams upstream, which is why it was chosen to infer its natal origin using the Sr isotope ratio in the otolith core [47]. The resulting information can be used to identify threats to the migration pathways due to dam construction or habitat changes.

The primary aim of the present study was to identify *P*. *krempfi* spawning grounds and the possible effects of habitat changes (such as dam construction) on this anadromous species. Our specific objectives were to (i) map the Sr isotope ratios (^87^Sr/^86^Sr) in waters along the Mekong River until Laos, and in some of its main tributaries, (ii) measure the concentrations of trace elements in the waters to identify differences between locations along the river, (iii) estimate the natal origin and migration behavior of *P*. *krempfi* caught in the Mekong Delta. Strontium isotope ratios in the water have never been recorded along the Mekong River for the purpose of tracing migrations. This catfish species was selected as a model because of its particular migratory behavior, and Sr isotope ratios in the water and otoliths were tested as tools for understanding fish migration. This research could provide fundamental data, including strontium isotope values in the Mekong and tributaries, to assign natal origin to larvae, and information on the migration pathway of other fish species caught in the Mekong basin using these isotopes.

## Materials and methods

### Study area

**Fig 1:**
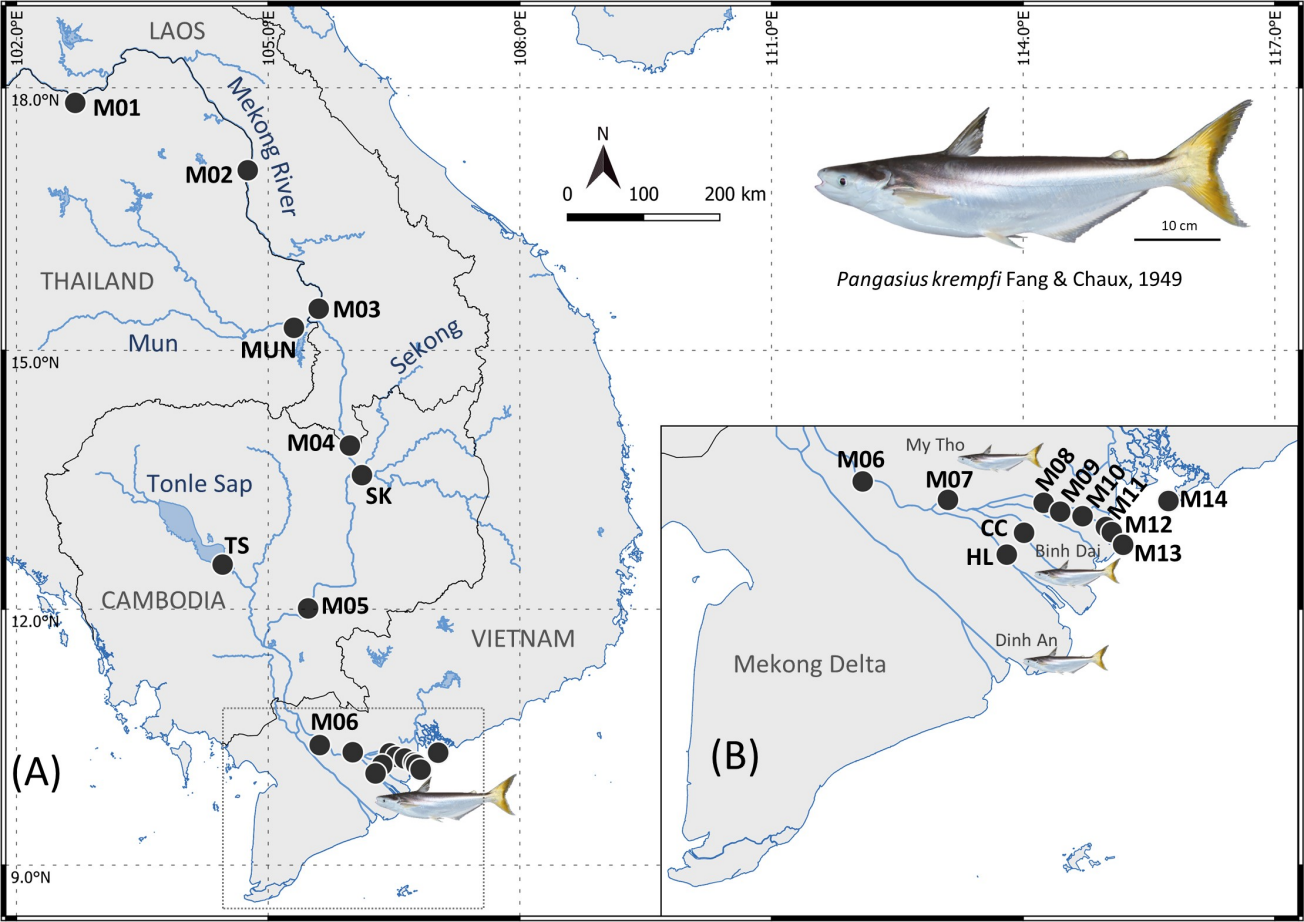
Sampling sites along the Mekong River. (A) Map of sampling sites for water (black circle) and fish (catfish) along the Mekong River, and (B) detailed map of the sites in the Mekong Delta.

The Mekong River rises in the Tibetan Plateau in China, and crosses Myanmar, Laos, Thailand, Cambodia, and Vietnam where it forms a gigantic delta before flowing into the sea though nine river mouths. From Laos, the mainstream is supplied by water flowing from three main complex tributaries, Chi-Mun River and Tonle Sap Lake on the right bank, and San-Srepok Rivers that form the Sekong River on the left bank (Fig 1A) [48]. After crossing Phnom Penh city (Cambodia), the Mekong River turns into two distributaries, the Mekong-Tien River and the Bassac-Hau River, which, in Vietnam, form a massive delta that drains into the sea through nine river mouths (Fig 1B) [49].

This study was conducted in the large stretch of the Mekong River that crosses four countries (Fig 1), Laos, Thailand, Cambodia, and Vietnam. The 19 sample locations were evenly spaced from the mouth of the river up to a site close to Vientiane, the capital of Laos (Fig 1 and Table 1). The upstream sampling sites began along the Thailand bank of the river in Laos to Cambodia and continued to downstream sites in the Mekong Delta in Vietnam where they were confirmed as representative of the salinity gradient (Table 1). Fourteen sampling sites belonged from the Mekong mainstream (M01 to M14), three on tributaries (Mun River—MUN, Sekong River—SK, and Tonle Sap—TS), and two on distributaries in the Mekong Delta (Co Chien River—CC and Ham Luong River–HL, in the Tien River), were sampled between April 2018 and September 2019.

**Table 1:** Water samples collected along the Mekong River and its delta.

Site	Location	Coordinates		Sampling date	Salinity	Distance from the sea (km)
		Latitude	Longitude			
**M01**	Nong Khai, Thailand	17°49.740’N	102°41.878’E	September 2019	0.0	1,529
**M02**	Nakhon Phanom, Thailand	17°3.735’N	104°45.268’E	September 2019	0.0	1158
**M03**	Ubon Ratchathani, Thailand	15°23.823’N	105°32.737’E	September 2019	0.0	903
**M04**	Khone Fall, Stung Treng, Cambodia	13°34.815’N	106°0.178’E	August 2019	0.0	646
**M05**	Kampong Cham, Cambodia	12°0.442’N	105°28.461’E	June 2019	0.0	417
**M06**	Dong Thap, Vietnam	10°24.514’N	105°36.764’E	May 2019	0.0	143
**M07**	Tien Giang, Vietnam	10°19.488’N	106°0.396’E	May 2018	0.1	90
**M08**	Chau Thanh, Ben Tre, Vietnam	10°18.699’N	106°26.909’E	April 2018	1.0	39
**M09**	Binh Dai, Ben Tre, Vietnam	10°16.266’N	106°31.494’E	April 2018	5.9	29
**M10**	Binh Dai, Ben Tre, Vietnam	10°15.048’N	106°37.698’E	April 2018	10.7	18
**M11**	Binh Dai, Ben Tre, Vietnam	10°12.114’N	106°44.166’E	April 2018	16.6	5
**M12**	Binh Dai, Ben Tre, Vietnam	10°10.703’N	106°45.793’E	April 2018	21.3	2
**M13**	Binh Dai, Ben Tre, Vietnam	10°7.254’N	106°48.948’E	April 2018	26.7	0
**M14**	Vung Tau, Vietnam	10°19.188’N	107°1.494’E	May 2018	30.0	0
**MUN**	Mun River, Ubon Ratchathani, Thailand	15°18.133’N	105°28.527’E	September 2019	0.0	869
**SK**	Sekong River, Stung Treng, Cambodia	13°32.735’N	106°0.875’E	June 2019	0.0	646
**TS**	Tonle Sap, Kampong Thom, Cambodia	12°31.099’N	104°27.334’E	June 2019	0.0	443
**HL**	Ham Luong River, Ben Tre, Vietnam	10°10.518’N	106°21.480’E	May 2018	0.1	41
**CC**	Co Chien River, Ben Tre, Vietnam	10°4.561’N	106°16.693’E	May 2018	0.1	41

Sampling locations and date, in situ salinity and distance from the sea are given. Salinity was measured directly in the field using a Castaway® CTD.

Sampling was authorized in Vietnam by the Fisheries Department (Ben Tre Province) for restricted areas. In Cambodia and Thailand, the authors were directly involved in the study, covering the permission required.

### Water sampling and analyses

At each sampling site, two replicates of 50 ml surface water were filtered through Minisart® cellulose acetate filters (pore size 0.2 μm, to remove suspended solids) into polypropylene tubes previously rinsed with pure nitric acid and MilliQ water. Each sample was fixed with 0.2 ml of 0.2% ultra-pure nitric acid. Salinity was measured directly in the field at the same time as the sampling using a Castaway® CTD probe.

To cross-validate the analytical methods, one of each replicate was analyzed in different laboratories. Two types of analysis were carried out on each replicate: trace element concentrations and the strontium isotope ratio (^87^Sr/^86^Sr). The trace element concentrations (^7^Li, ^24^Mg, ^31^P, ^55^Mn, ^66^Zn, ^85^Rb, ^88^Sr, ^118^Sn, ^138^Ba and ^208^Pb) of one replicate from each sampling site were analyzed at the AETE-ISO OREME laboratory (*Analyse des Eléments en Trace dans l’Environnement & ISOtopes–Observatoire de REcherche Méditerranéen de l’Environnement*, University of Montpellier, Montpellier, France) using solution-based inductively coupled plasma mass spectrometry (ICP-MS). Then, the same replicates were analyzed at the SARM laboratory (*Service d’Analyse des Roches et des Minéraux—Centre de Recherches Pétrographiques et Géochimiques*, Nancy, France) to measure Sr isotopes (^87^Sr and ^86^Sr) using multi-collector inductively coupled plasma mass spectrometry (MC-ICP-MS). The other replicates were analyzed at IES (Institute of Earth Science, Academia Sinica, Taiwan) to measure both the trace element concentrations (^7^Li, ^11^B, ^23^Na, ^24^Mg, ^27^Al, ^28^Si, ^39^K, ^40^Ca, ^55^Mn, ^63^Cu, ^66^Zn, ^88^Sr, ^138^Ba, ^208^Pb, and ^238^U), using high resolution inductively coupled plasma mass spectrometry (HR-ICP-MS), and the Sr isotope ratio, using high-resolution multiple collector inductively-coupled plasma mass spectrometry (HR-MC-ICP-MS). A summary of the analytical procedures is provided in (S1 Fig).

The AETE-ISO OREME laboratory measured trace element concentrations using solution-based ICP-MS, (7700x; Agilent, Santa Clara, CA, USA) as described in Tran et al. [47]. Samples were filtered with 0.22 mm mesh size to remove suspended particles and diluted by a factor between two and four, depending on their measured salinity, using milli-Q water to ensure that the concentrations of trace elements to be detected were within equipment’s measurement limits. Indium (^115^In) and Bismuth (^209^Bi) were used as internal standards. Concentrations were determined by external calibration using multi-element standard solutions with concentrations in the range 0.25–5 ppb. Polyatomic interference was limited by keeping the oxide level below 1%. The certified material SLRS-6 (National Research Council Canada, Ottawa, ON, Canada) was used as standard, and analytical precisions were generally between 1 and 3% relative standard deviation (RSD).

The SARM laboratory measured the Sr isotope ratios using thermal ionization mass spectrometry (TIMS, ThermoFisher Scientific TRITON Plus). Water samples were evaporated and 1.5–2.0 μg of Sr was loaded onto chromatographic separation columns with a Sr Spec specific resin (modified after Pin et al. [50]) for separation. The Sr was then loaded onto rhenium filaments and measured with a ThermoFisher Scientific TRITON Plus. The strontium isotopic ratios are expressed as ^87^Sr/^86^Sr and were normalized to the stable ^86^Sr/^88^Sr ratio of 0.1194 to correct for instrumental mass fractionation. Instrument accuracy was assessed by repeated analyses of the standard reference material NIST SRM 987 (National Institute of Standards and Technology, USA), and the blank values were below 300 pg.

The IES laboratory measured the trace element concentrations using HR-ICP-MS (ThermoFisher Scientific, Element XR). As there was a wide range of salinities in the water samples, thus causing matrix effects, the saline waters collected from the estuary and coastal areas were diluted to Na concentration of 10 ppm (around 0.03 salinity) by adding 0.1N HNO_3_. The freshwater samples were analysed directly. Six in-house standards were prepared with high-purity single element products (high purity standard, U.S.) in a series of different element concentrations and these standards were used to establish calibration curves. A total of 15 trace element concentrations were measured. The analysis of SRM 1640a (the reference material for trace elements in natural water, National Institute of Standards and Technology, USA) resulted in trace element concentrations with an RSD compared to the certified values of better than 10% for all trace elements except Si, Ba and Mn (data in S1 Table). Repeated analysis of SRM 1640a gave a measurement precision better than 0.35 (1 standard deviation - 1SD) in all cases.

The IES laboratory measured the Sr isotope ratios using HR-MC-ICP-MS (ThermoFisher Scientific, Neptune Plus). The Sr element was purified using the procedure developed by Huang et al. [51] and Huang and You [52]. Water samples were passed through Sr resin (Eichrom^TM^) and Sr was then eluted with 0.05N HNO_3_. The elutant was slowly heated on a hot plate until completely dry. The dried samples were re-dissolved in 0.1M HCl and then diluted to a consistent Sr concentration of 10 ppb for isotope analyses. The blank was less than 5 pg for Sr and the whole procedure with the IAPSO Seawater Standard revealed 100% recovery of Sr in long-term tests. The sample standard bracketing method was applied to determine the Sr isotope value of the water samples, and the certified international standard, SRM 987 was used as a reference. IAPSO Seawater Standard was also treated as an unknown sample for quality and quantity control, yielding high accuracy (RSD 0.001%) and precision (1SD 4.2 × 10^−6^).

### Otolith sampling and microchemical analysis

A total of 27 individual *Pangasius krempfi* (47.8 to 84.5 cm total length) were collected from March-August 2017 (24 individuals) and in April 2018 (3 individuals) in the Mekong Delta. The fish were collected on landing or in markets in Binh Dai city (18 individuals, coded BD01 to BD18), My Tho city (7 individuals, MT01 to MT07) and Dinh An city (2 individuals, DA01 and DA02) (Fig 1). Samples were collected randomly to get a wide range of lengths and weights. The left (the larger) otolith (*lapillus*) was extracted using plastic forceps from a sagittal head section, cleaned in Milli-Q water, and stored dry in 1.5 ml microtubes. As the right otolith had been used in another study [47], the age and Sr/Ca ratio of the individuals were already known.

The otoliths were prepared at the University of Science, VNU-HCM (Vietnam). They were first photographed whole under reflected light, then embedded in epoxy resin (Creative Life Vietnam, Vietnam), sliced transversally using a Buehler IsoMet Low Speed cutting machine (Buehler, Germany) and polished until the core was reached (S2 Fig). Sections were then attached to a clean microscope slide for further processing. Sr isotopes were measured at the AETE-ISO OREME lab, Montpellier, France) using a Thermo Finnigan Neptune^+^ multicollector inductively-coupled plasma mass spectrometer (MC-ICP-MS) coupled to a 193 nm Analyte G2 Excimer Laser Ablation system (Photon Machines Inc). Spots were ablated at 65 μm diameter at 5μm/s. The pulse rate was 7 Hz and the energy density of the beam was 6.0 J/cm^2^. A pre-ablation transect (spot size 85 μm, speed 15 μm/s) was used to clean the sample surface before analysis. A typical analysis comprised a 30 s background measurement and the ablation period required for a transect from the core to the edge. Corrections for Kr and Rb interference and mass bias followed routine procedures using known isotopic ratios [53]. No corrections were applied for interference from doubly charged REE, Ca argides and Ca dimers and polyatomic interference, since numerous studies have shown that Ca argides and dimers have no significant effect on Sr isotopic data using MC-ICP-MS [54]. Ca-P-O and doubly charged ions are also insignificant for material with a high Sr (Sr > 300ppm) and low REE contents. Krypton interference (^84,86^Kr on ^84,86^Sr) from the argon tank was corrected for by measuring the background level before the analysis, and then by subtracting the background from the data. Rubidium interference (^87^Rb on ^87^Sr) was corrected for by monitoring ^85^Rb and subtracting the signal at mass 87 amu assuming a natural ^85^Rb/^87^Rb of 2.59262 [55]. The ^85^Rb/^87^Rb ratio was corrected for mass bias using the mass discrimination factor calculated from Sr, using an exponential law and a natural ^88^Sr/^86^Sr = 8.375209, and assuming no differential mass discrimination between Sr and Rb. The accuracy and long-term reproducibility of the measurements were checked by analyzing the pressed pellet MACS3 from the USGS (United States Geological Survey, Reston, VA, USA; reference value of ^87^Sr/^86^Sr = 0.70755) and in-house reference material (Atlantic shell with a reference value of ^87^Sr/^86^Sr = 0.70918).

### Statistical analyses

All data were tested for normality using the Shapiro-Wilk test [56], and for homoscedasticity using Levene’s test prior to using parametric or non-parametric tests. Due to the absence of normality in trace element concentrations, differences in measurement precision and accuracy between the laboratories were compared using the Wilcoxon test.

The ^86^Sr/^87^Sr values of the two replicate sets of water samples were normally distributed and were compared using a paired Student’s *t-*test. The relationship between these values was calculated using a general linear regression. The correlation between ^87^Sr/^86^Sr and water salinity, the distance from the sea and 1/Sr were tested using a Pearson test, and the relationships were then tested using linear or logarithmic regressions based on the R^2^ value.

For each sampling site, the mean of the concentrations for each trace element obtained by the two laboratories was used for subsequent analyses. The concentrations of trace elements that were only measured by one laboratory were used directly. Correlations between trace element concentrations and salinity in water were tested by using a Pearson correlation test for Mg and Spearman correlation test for other trace elements. Concentrations of ^28^Si, ^63^Cu, ^138^Ba and ^238^U were only detected in freshwater and were not used to test correlations with salinity. Among these trace elements, only the Sr/Ca and Ba/Ca ratios were tested to determine if they were able identify differences in water chemistry between locations, as mentioned by Fukushima et al. [11].

The ^87^Sr/^86^Sr profiles were calculated for each otolith using moving means of three adjacent values at a given distance from the core to the edge. ANOVA was used to test for differences in otolith ^87^Sr/^86^Sr between fish sampled at different locations (Binh Dai, My Tho and Tien Giang), or between years (fish collected in 2017 and 2018). To test for similarity of ^87^Sr/^86^Sr profiles between individuals and for distinct groups of *P*. *krempfi* based on ^87^Sr/^86^Sr profile, a hierarchical cluster analysis (HCA) was used based on the Euclidean distances between ^87^Sr/^86^Sr profiles and Ward’s clustering method (“ade4” package in R). The number of clusters was determined based on the performance of hierarchical cluster analysis. ^87^Sr/^86^Sr profiles were also compared by direct observation to the Sr/Ca ratio already available from the right otolith in a previous publication [47]. All statistical analyses were performed in R [57].

### Ethics statement

All the fish samples used in this study were collected dead at fish landings from fishermen or in markets. Thus, all fish were dead at sampling and no ethical approval is required. The sampling in Vietnam was hosted by University of Science–VNUHCM with the permission from Ben Tre Fishery Department and Immigration Department (number 317/KHTN-QHQT-QLDA on 12/04/2018). Other sampling locations in Cambodia and Thailand are not included in protected areas or restricted areas and there is no requirement for official permission.

## Results

### Strontium isotope ratios (^87^Sr/^86^Sr) and trace element concentrations in the water

**Fig 2:**
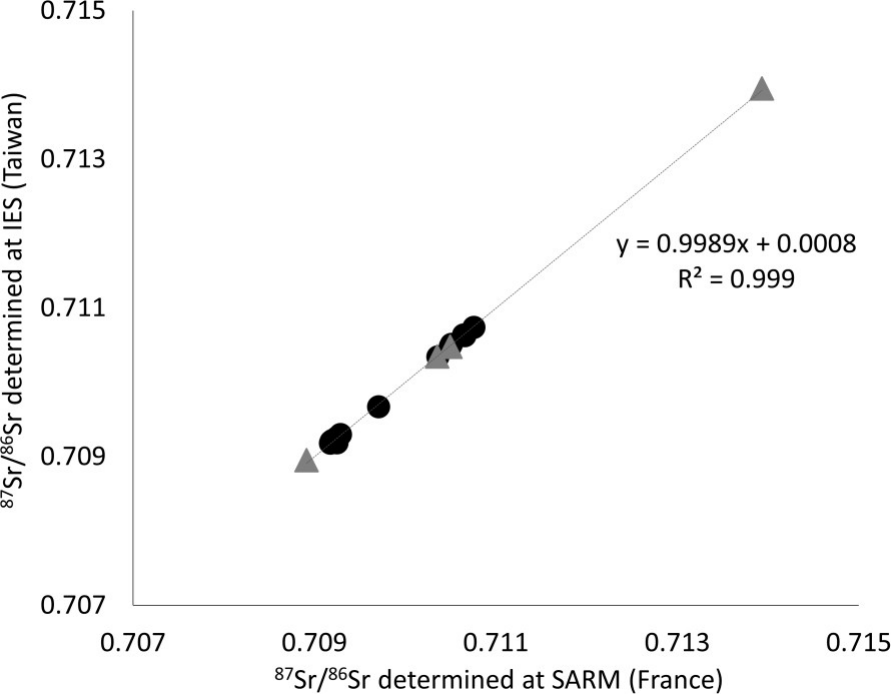
Relationship between ^87^Sr/^86^Sr determined by the two laboratories (AETE-ISO OREME and IES). Linear regression between the ^87^Sr/^86^Sr in water samples reported by IES (Taiwan) versus those reported by SARM (France). Black circles indicate sites along the Mekong mainstream, grey triangles indicate sites on tributaries and distributaries.

The differences in ^87^Sr/^86^Sr between SARM France and IES Taiwan were well within 2σ, being less than 0.000022 (Table 2) and were not statistically significant (paired Student’s *t-*test, t = 0.347, df = 18, p-value = 0.732). The relationship between ^87^Sr/^86^Sr in water analyzed at the two laboratories was linear and highly significant (Fig 2; R^2^ = 0.999, slope = 0.999). For each sampling site, the mean of the ^87^Sr/^86^Sr from the two laboratories was used for subsequent analyses.

**Table 2:** ^87^Sr/^86^Sr and Sr concentrations.

Sampling Site	^87^Sr/^86^Sr			Sr (ppb)		
	SARM (France)	IES (Taiwan)	Mean ±SD	AETE-ISO OREME (France)	IES (Taiwan)	Mean ±SD
**M01**	0.710646	0.710638	0.710642 ± 4 × 10^−6^	149.08	123.04	136.06 ± 13.02
**M02**	0.710656	0.710632	0.710644 ± 12 × 10^−6^	99.71	83.05	91.38 ± 8.33
**M03**	0.710757	0.710742	0.710750 ± 7 × 10^−6^	82.87	64.03	73.45 ± 9.42
**M04**	0.710639	0.710641	0.710640 ± 1 × 10^−6^	50.61	45.12	47.86 ± 2.74
**M05**	0.710358	0.710345	0.710352 ± 6 × 10^−6^	178.75	151.96	165.36 ± 13.39
**M06**	0.710495	0.710485	0.710490 ± 5 × 10^−6^	157.95	89.03	123.49 ± 34.46
**M07**	0.710507	0.710515	0.710511 ± 4 × 10^−6^	161.74	114.00	137.87 ± 23.87
**M08**	0.709707	0.709672	0.709690 ± 17 × 10^−6^	335.75	331.00	333.38 ± 2.38
**M09**	0.709284	0.709303	0.709293 ± 9 × 10^−6^	1398.54	1431.00	1414.77 ± 16.23
**M10**	0.709246	0.709259	0.709253 ± 7 × 10^−6^	2030.00	2288.00	2159.00 ± 129
**M11**	0.709197	0.709221	0.709209 ± 12 × 10^−6^	2464.69	4196.00	3330.35 ± 865.65
**M12**	0.709246	0.709187	0.709216 ± 30 × 10^−6^	8369.77	5132.00	6750.88 ± 1618.88
**M13**	0.709174	0.709187	0.709180 ± 6 × 10^−6^	6432.31	6266.00	6349.15 ± 83.15
**M14**	0.709182	0.709209	0.709195 ± 13 × 10^−6^	6738.53	7340.00	7039.26 ± 300.74
**MUN**	0.710353	0.710350	0.710351 ± 2 × 10^−6^	32.60	26.03	29.32 ± 3.29
**SK**	0.713932	0.713955	0.713943 ± 11 × 10^−6^	34.79	33.20	33.99 ± 0.79
**TS**	0.708914	0.708954	0.708934 ± 20 × 10^−6^	46.73	43.16	44.95 ± 1.79
**HL**	0.710499	0.710477	0.710488 ± 11 × 10^−6^	149.13	94.00	121.56 ± 27.56
**CC**	0.710494	0.710477	0.710486 ± 8 × 10^−6^	156.71	142.00	149.35 ± 7.35

^87^Sr/^86^Sr and Sr concentrations (mean ± S.D.) measured by AETE-ISO OREME and SARM (France) compared with those measured by IES (Taiwan).

The ^87^Sr/^86^Sr for water samples from the Mekong River ranged from 0.708934 ± 20 × 10^−6^ to 0.713943 ± 11 × 10^−**6**^ (Table 2). The ^87^Sr/^86^Sr for the Mekong mainstream ranged from 0.709180 ± 6 × 10^−6^ at the sea to 0.710750 ± 7 × 10^−6^ upstream. In the tributaries, the ^87^Sr/^86^Sr were lower in the Tonle Sap Lake (0.708934 ± 20 × 10^−6^) and higher in the Sekong River (0.713943 ± 11 × 10^−6^) which was higher than any of the ratios in the Mekong mainstream. The other tributary (Mun River–MUN) and the distributaries (Ham Luong—HL and Co Chien River—CC) had ^87^Sr/^86^Sr of 0.710351 ± 2 × 10^−6^, 0.710488 ± 11 × 10^−6^ and 0.710486 ± 8 × 10^−6^ respectively, similar to the upper part of Mekong mainstream.

**Fig 3:**
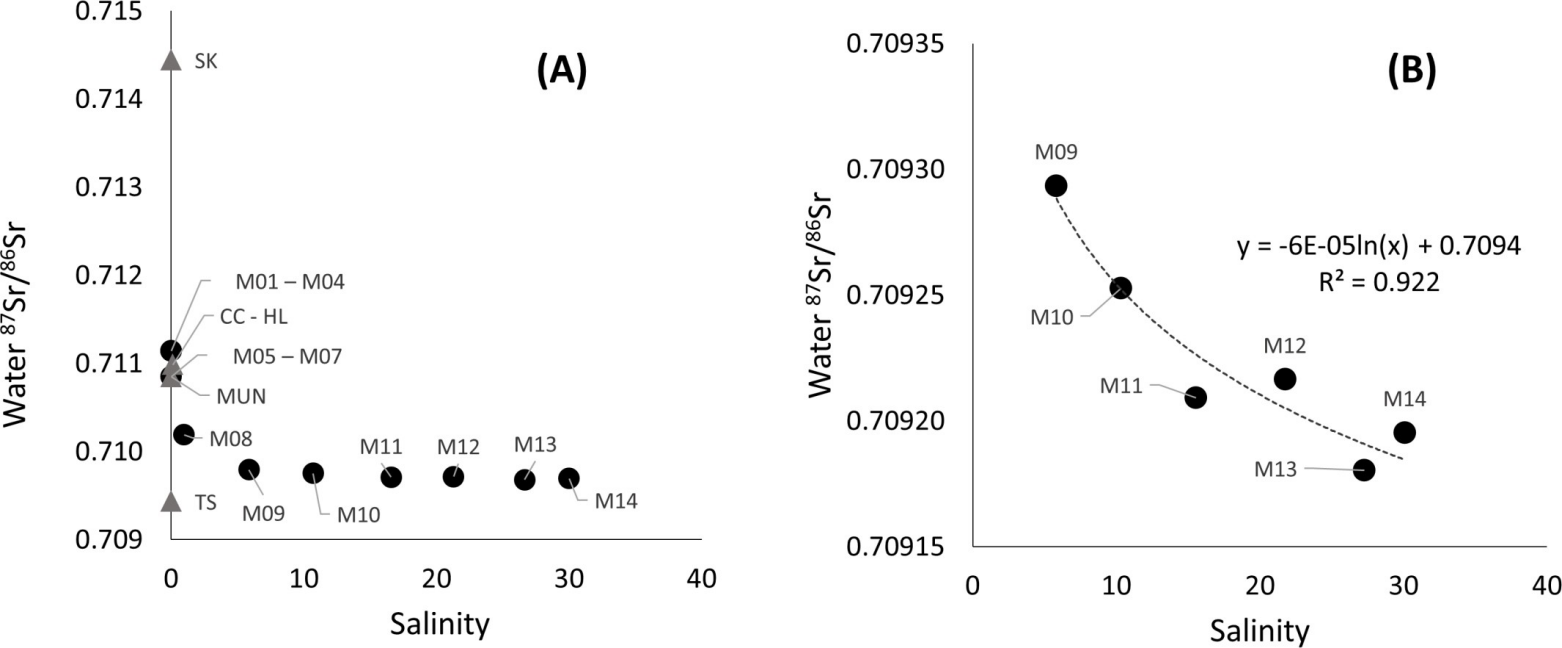
Water ^87^Sr/^86^Sr versus salinity along the Mekong River. Water ^87^Sr/^86^Sr versus salinity along the Mekong mainstream (dark circles) and tributaries / distributaries (grey triangles); (A) for all sampling locations, and (B) only in saline waters (Mekong Delta) with a logarithmic regression. Labels at each point refer to the sampling sites in Fig 1 and Table 1.

**Fig 4:**
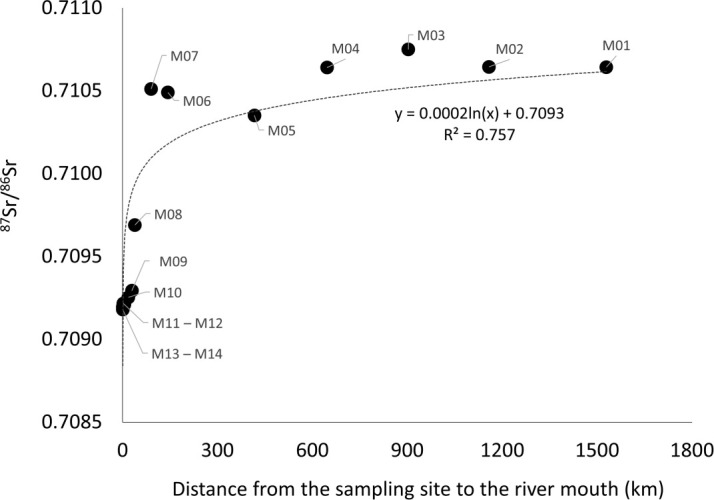
Water ^87^Sr/^86^Sr ratio versus distance from the sea along the Mekong River. Water ^87^Sr/^86^Sr versus distance from the sea along the Mekong mainstream. Distances were estimated using Google Earth Pro. Labels at each point refer to the sampling sites in Fig 1 and Table 1.

**Fig 5:**
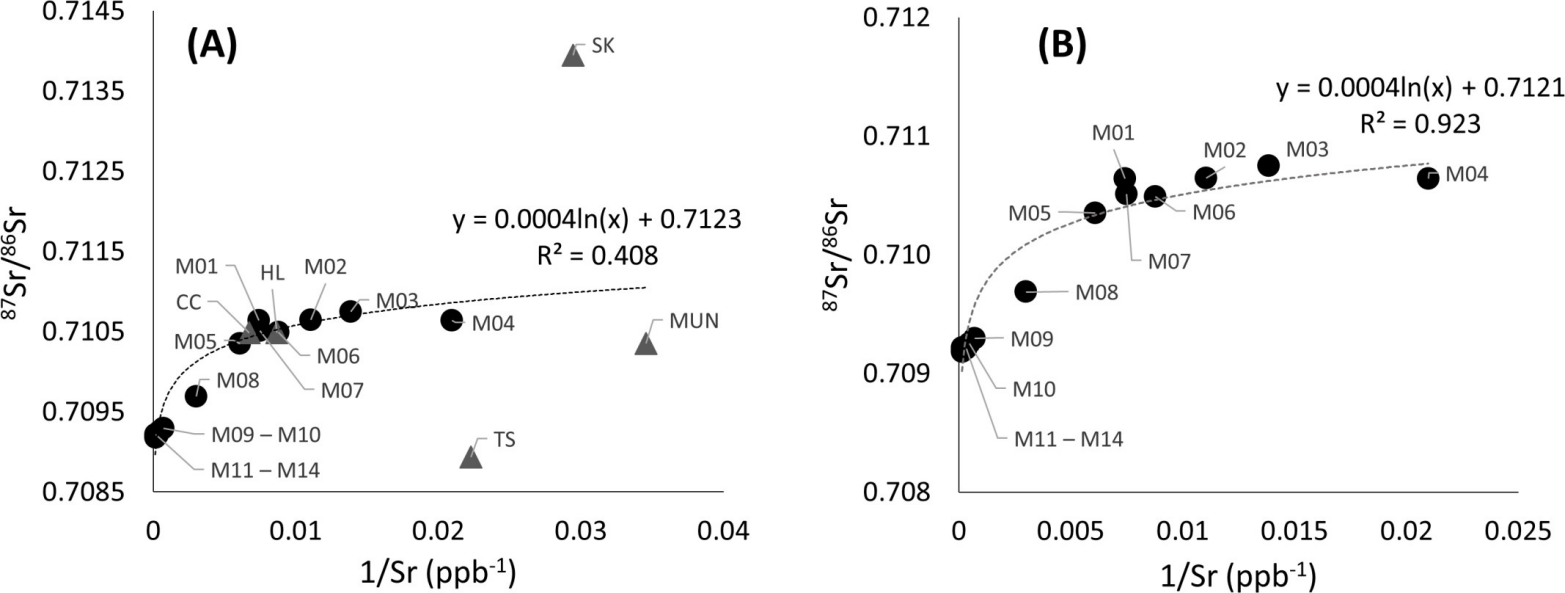
Water ^87^Sr/^86^Sr against 1/Sr. Water ^87^Sr/^86^Sr versus 1/Sr along the Mekong River; (A) with and (B) without tributaries and distributaries. Labels at each point refer to the sampling sites in Fig 1 and Table 1. Black circles indicate sites on the Mekong mainstream, grey triangles indicate sites belonging on tributaries and distributaries.

The ^87^Sr/^86^Sr was negatively correlated with salinity (Pearson correlation = - 0.523, p-value = 0.021). The ^87^Sr/^86^Sr was always much higher in freshwater (Fig 3A) while there was a significant logarithmic regression between ^87^Sr/^86^Sr and salinity in more saline waters (R^2^ = 0.922, Fig 3B). The ^87^Sr/^86^Sr was non-significantly correlated with the distance from the sampling location to the sea (Pearson correlation = 0.449, p-value = 0.053), but there was a significant logarithmic regression between these two variables along the Mekong mainstream (R^2^ = 0.757, Fig 4). The ^87^Sr/^86^Sr was divided into five groups depending on their value, salinity and distance from the sea: a saline and brackish water region in Mekong Delta (sites M09 to M14), a freshwater region in the Mekong Delta (site M05 to M08, CC, and HL), upstream regions (sites M01 to M04 and Mun), the Sekong River and Tonle Sap Lake. The relationship between concentrations of ^87^Sr/^86^Sr and 1/Sr in the water was more exponential (R^2^ = 0.408), but not significant, when all sites were included, the relationship was exponential only if the tributaries and distributaries were excluded (R^2^ = 0.923, Fig 5), and the correlation between ^87^Sr/^86^Sr and 1/Sr was significant (Pearson correlation = 0.592, p-value = 0.007).

The concentrations of ^31^P, ^28^Si, ^63^Cu, and ^238^U in brackish and marine water samples were below the limit of detection, and were therefore neglected in further analyses. The trace element concentrations did not differ between two analytical methods used in AETE-ISO OREME and IES (Table 3, Wilcoxon test, p-value > 0.05). Most of the trace element concentrations (^7^Li, ^11^B, ^23^Na, ^24^Mg, ^39^K, ^40^Ca, ^66^Zn, ^85^Rb and ^88^Sr) were significantly positively correlated with water salinity (Spearman’s correlation, p-value < 0.05), when ^27^Al, ^31^P, ^55^Mn, ^118^Sn, ^138^Ba, ^208^Pb showed no significant correlation (Spearman’s correlation and Pearson correlation, p-value > 0.05) and ^28^Si, ^63^Cu, ^138^Ba, ^238^U only detected in freshwater region (Table 3). The value of water salinity and trace element concentrations are available in S2 Table.

**Table 3:** Trace element concentrations measured by the two laboratories (AETE-ISO OREME and IES).

Trace element	Number of samples *measured by AETE-ISO OREME*	Number of samples *measured by IES*	Difference between two labs (p-value)	Correlation with water salinity	Comments
				(* = p > 0.05)	
^**7**^**Li**	19	19	0.080	0.829	
^**11**^**B**		19	-	0.943	
^**23**^**Na**		19	-	0.945	
^**24**^**Mg**	9	19	0.129	0.999	Value from AETE-ISO OREME only outside the Mekong Delta
^**27**^**Al**		19	-	0.431*	
^**28**^**Si**		13	-	-	Value only from freshwater regions
^**31**^**P**	6	-	-	0.410*	Only a few samples were detected
^**39**^**K**		19	-	0.931	
^**40**^**Ca**		19	-	0.879	
^**55**^**Mn**	19	11	0.175	-0.042*	
^**63**^**Cu**		12	-	-	Value only from freshwater regions
^**66**^**Zn**	19	11	0.365	0.596	
^**85**^**Rb**	19	-	-	0.886	
^**88**^**Sr**	19	19	0.197	0.883	
^**118**^**Sn**	19	-	-	-0.229*	
^**138**^**Ba**	19	13	0.070	-0.254*	Value from IES only from freshwater regions
^**208**^**Pb**	19	-	-	0.410*	
^**238**^**U**		11	-	-	Value only from freshwater regions

Trace element concentrations measured by the two laboratories (AETE-ISO OREME and IES), with p-value (Wilcoxon test) for differences between the two laboratories and correlation between trace element concentrations in the water vs. salinity (Spearman’s correlation and Pearson correlation). * significant correlation (p > 0.05).

**Fig 6:**
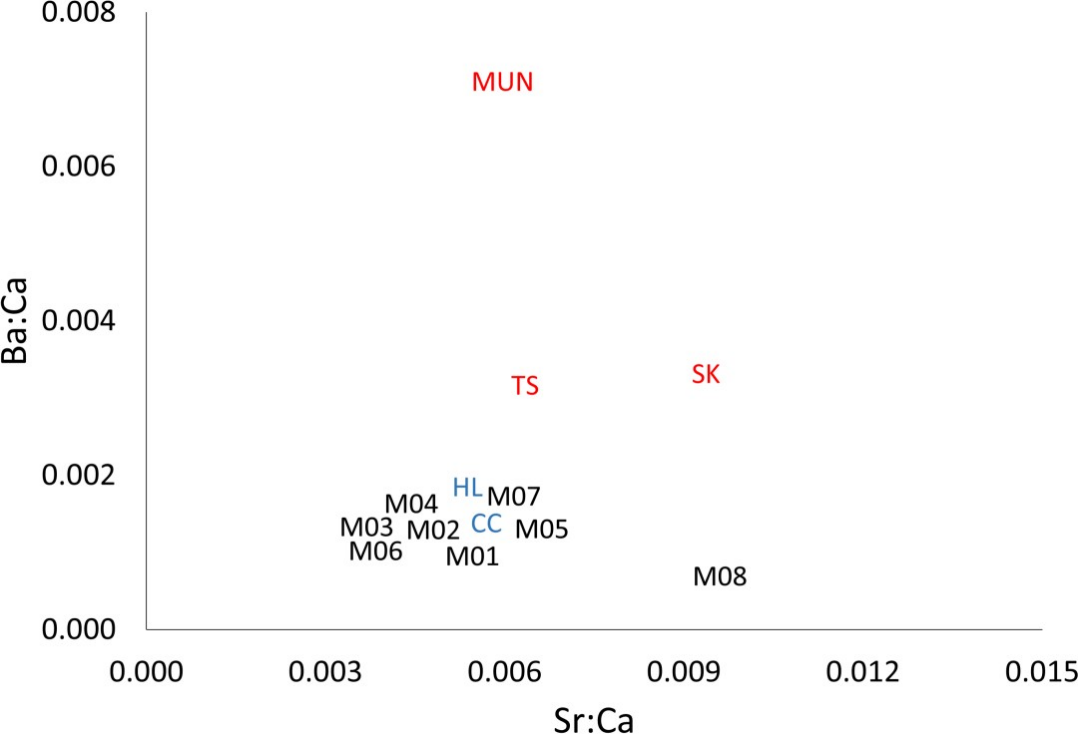
Ba/Ca ratios versus Sr/Ca ratios in the Mekong mainstream and tributaries. Labels at each point refer to the sampling sites in Fig 1 and Table 1. Black letters correspond to sites on the Mekong mainstream, red letters to sites on tributaries, and blue letters to sites on distributaries.

Correlations between trace element concentrations and salinity were highly significant and positive for ^7^Li, ^11^B, ^23^Na, ^24^Mg, ^39^K, ^40^Ca, ^85^Rb and ^88^Sr, whereas for ^66^Zn the correlation was less significant, and no correlation was found for ^19^Sn, ^27^Al ^55^Mn, ^138^Ba and ^208^Pb (Table 3 and S3 Fig). The relationships between Sr/Ca ratios and Ba/Ca ratios were quite close in the freshwater parts of the Mekong mainstream compared to values in tributaries (MUN, SK and TS) (Fig 6). Other trace elements showed no or only slight differences among sites indicating their limitations as biotracers in the Mekong mainstream.

### Strontium isotope ratios in *Pangasius krempfi* otoliths

**Fig 7:**
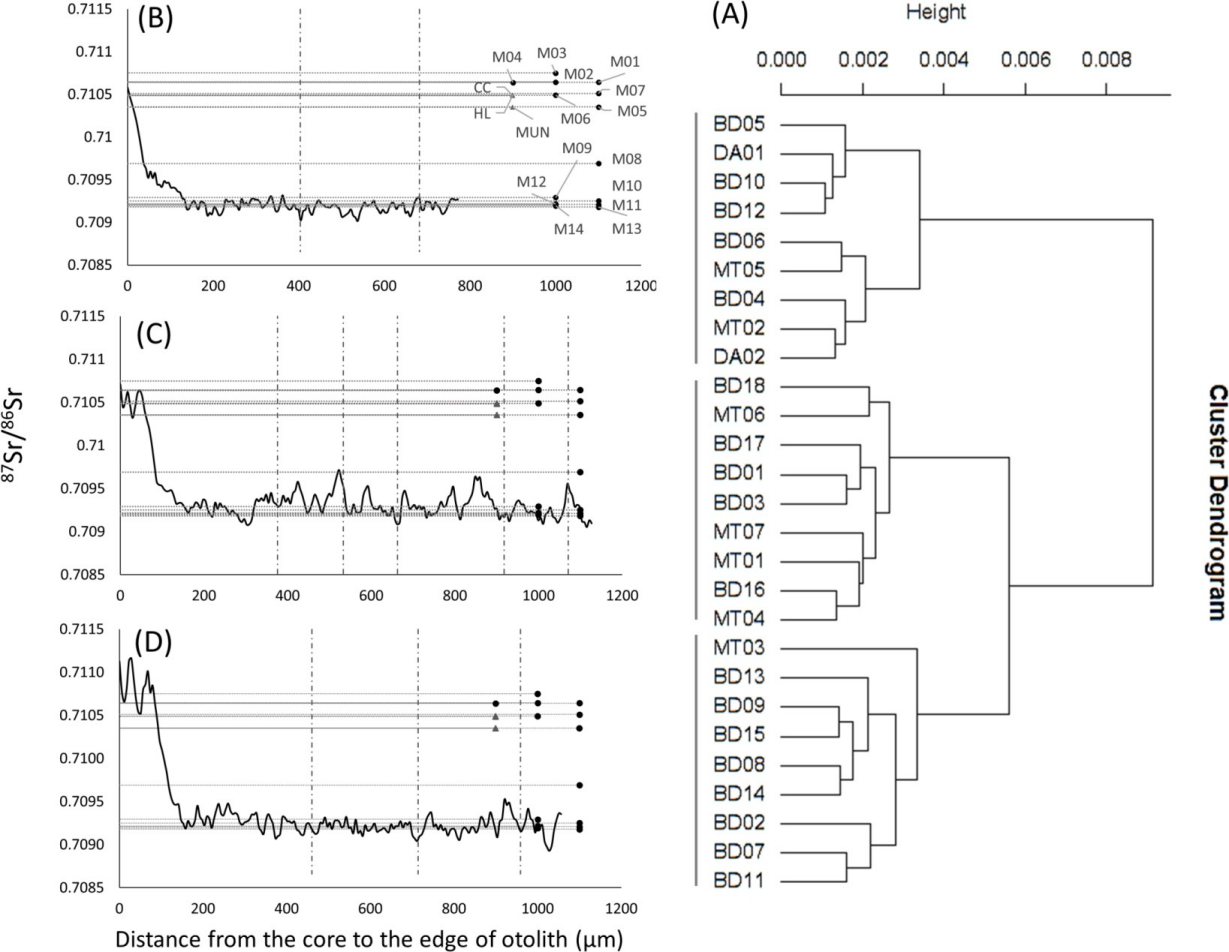
Main patterns of ^87^Sr/^86^Sr profiles in the otoliths of *P*. *krempfi* caught in the Mekong Delta. (A) Dendrogram of the hierarchical cluster analysis on the right. (B, C and D) on the left, three main types of ^87^Sr/^86^Sr profiles from the core to the edge of the otolith coming from the three branches of the dendrogram. Vertical dotted lines mark the end of each year based on the position of the annual growth increments. Horizontal lines correspond to the mean water ^87^Sr/^86^Sr at each sampling location in the Mekong River, except for sampling locations in the Sekong River and Tonle Sap Lake that had higher and lower values, respectively. Labels on the point on each horizontal line refer to the sampling sites (Fig 1 and Table 1). Black dots indicate Mekong mainstream sites; grey triangles indicate a tributary or distributary.

All the otoliths showed similar ^87^Sr/^86^Sr patterns over the lifetime of the fish (Fig 7 and S3 Table): high values at hatching and during the early days of life (0.7106 ± 0.0003 at the core region ~ 30 μm width), corresponding to the ^87^Sr/^86^Sr of waters collected upstream along the Laos–Thailand border and through Cambodia (sites M01 to M04). The ratios then decreased to a more or less variable plateau (0.70928 ± 7.4 × 10^−6^), corresponding to the ^87^Sr/^86^Sr of the brackish waters (site M09 at Binh Dai). Otolith ^87^Sr/^86^Sr never showed values corresponding to water samples collected from Tonle Sap Lake or the Sekong River. There was no difference in ^87^Sr/^86^Sr in the otoliths in fish sampled at locations Binh Dai (BD01 –BD18), My Tho (MT01 –MT07) and Dinh An (DA01 and DA02) in the Mekong Delta, or between years (BD16 –BD18 were collected in 2018 and the other samples were collected in 2017) (ANOVA, p-value = 0.47 and 0.75, respectively). Comparing the ratios 15 μm from the core with those in the water showed that all individuals were born in the Mekong mainstream and not in tributaries. Nevertheless, the differences in ^87^Sr/^86^Sr (Fig 7) at the core pointed to different spawning grounds and different behaviors during the early life stages. The lowest ^87^Sr/^86^Sr in the otoliths was lower than the lowest ^87^Sr/^86^Sr in saline water that was collected at Vung Tau (site M14), suggesting that the fish may move to saline areas other than our sampling locations once they reach brackish water.

The hierarchical cluster analysis of the general shape of otolith ^87^Sr/^86^Sr profiles produced three main clusters (Fig 7A). One cluster had lower ^87^Sr/^86^Sr at the core (1/3 of individuals), close to the ratios at sites M06 and M07 in the freshwater part of the Mekong Delta that fell to reach a plateau corresponding to the ^87^Sr/^86^Sr ratios in the saline waters at sites M10 to M14 in the part of the Mekong Delta where the water is brackish (Fig 7B). The second cluster had medium ^87^Sr/^86^Sr ratios at the core (1/3 of individuals), corresponding to the water ^87^Sr/^86^Sr encountered upstream in Thailand and Cambodia (sites M01 to M04), and variable ratios after reaching brackish water (sites M10 to M14, Fig 7C). In this cluster, after the fish had reached the Delta, the ^87^Sr/^86^Sr sometimes peaked close to the ratio found in the slightly brackish water at M08. The last cluster had higher, variable ratios at the core, higher than any recorded in the water in the Mekong mainstream (1/3 of individuals), falling to a plateau while the fish were in the brackish waters of the delta (ratios close to those at sites M11 to M14, Fig 7D).

**Fig 8:**
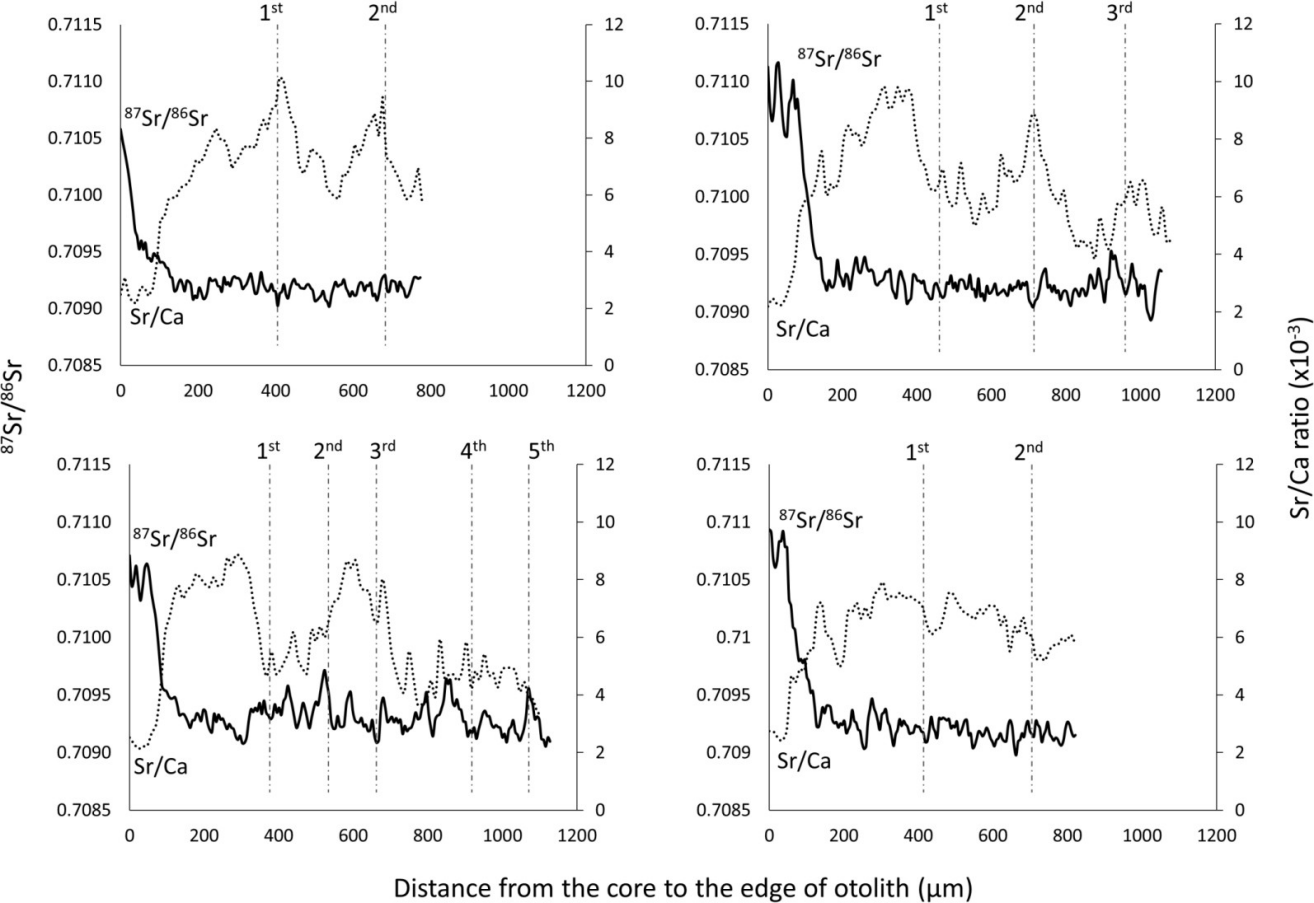
Comparison of ^87^Sr/^86^Sr profiles and Sr/Ca profiles for the same individuals. Comparisons of ^87^Sr/^86^Sr profiles (solid line) and Sr/Ca profiles (dotted line) for four *P*. *krempfi* individuals. Profiles are shown from the core to the edge of the transverse otolith section. Vertical dotted lines mark the end of each year based on the position of the annual growth increments.

In comparison, otolith Sr/Ca profiles measured in our previous study [47] had a very low ratio at the core, a rapid increase to higher ratios, and greater variations during the rest of the lifetime of the fish (Fig 8). While the ^87^Sr/^86^Sr profiles showed more variance close to the core, Sr/Ca profiles showed much more variation during the period of their life in brackish waters. Although the Sr/Ca ratio patterns along the otolith transect were the opposite of ^87^Sr/^86^Sr profiles, they showed the same migration patterns in the Mekong Delta with several peaks.

The patterns of all individuals suggest that they moved from freshwater at birth, down the Mekong mainstream to coastal, saline regions, during their lifetime. A large part of their lifetime was spent in the brackish and marine water regions in the Mekong Delta, where they were caught. After hatching, they spent only a few months, much less than 1 year (Fig 8), in the freshwaters of the Mekong in Thailand and Cambodia, before moving to the Mekong Delta. None of the individuals showed any evidence of having swum in the Mekong tributaries, such as the Mun River, the Sekong River, or Tonle Sap Lake.

## Discussion

In transboundary rivers, such as the Mekong River, migratory fish are threatened by climate changes, pollution, saline intrusions and the construction of dams [8–10]. Studying the movement patterns of migratory fish species is thus essential for fishery management and fish conservation across the Mekong countries [58, 59]. In this study, strontium isotope ratios (^87^Sr/^86^Sr) in the water and in fish otoliths were used to identify the migration pathways of one anadromous catfish, *Pangasius krempfi*, from the delta up to the Mekong River. The Sr isotope ratios and trace element concentrations in waters along the river and in some main tributaries were mapped in four countries (Vietnam, Cambodia, Thailand and Laos), to provide the data needed to estimate the origin and migration pathways of fish caught in the Mekong Delta.

### Strontium isotope ratios (^87^Sr/^86^Sr) and trace element concentrations in the water along the Mekong River

**Fig 9:**
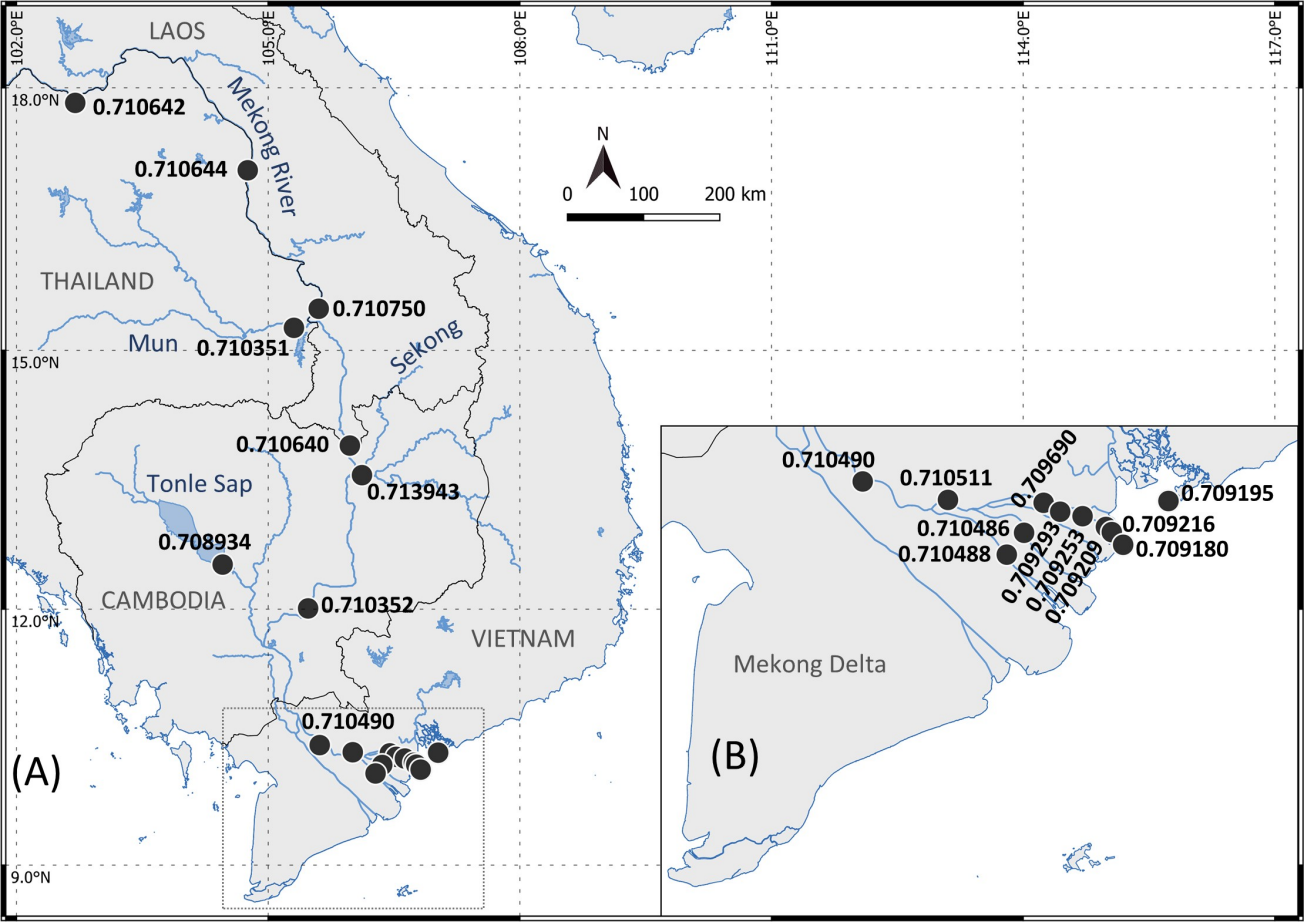
Map of ^87^Sr/^86^Sr values along the Mekong River and tributaries. Detailed values are listed in Table 2.

This is the first study mapping the ^87^Sr/^86^Sr ratios along the Mekong River, from the Laos-Thailand border to the delta and the sea, and in the main tributaries (Fig 9). The ^87^Sr/^86^Sr at the upper Mekong (0.71064) is below the average for big rivers (0.71107), reported by Peucker-Ehrenbrink et al. [60], and below the average of the Himalayan-Tibetan rivers (0.7127) reported by Richter et al. [61] but similar to that of the Amazon River (0.710–0.712) reported by Duponchelle et al. [40]. The ^87^Sr/^86^Sr at the Mekong mouth (0.70919) corresponds to the global average ^87^Sr/^86^Sr for seawater (0.70918, [38]). The strontium concentration and Sr isotope composition of rivers are largely induced by a mixture of strontium derived from the type of rocks encountered and their age [62, 63]. Therefore, the ^87^Sr/^86^Sr values measured from the upper Mekong to the mouth could reflect a mixture of sources in different geological regions along the Mekong River, including (1) Himalayan bedrocks in the upper Mekong, (2) the Annamite Range along the Laos–Cambodia—Vietnam border, and (3) primitive volcanic sources, or other tributaries in the Mekong Delta [64, 65]. Seawater, a source of strontium isotopes, could also affect the ^87^Sr/^86^Sr in the estuarine regions, as reported in a previous study [66].

The sites sampled in the mainstream (M01, M05) and in the delta (M06 to M14, CC, and HL) are influenced by quaternary sediments, while M02 and M03 are affected by shallow shelf-marine dating from the Cretaceous or Jurassic periods [65]. Concerning the tributaries, Tonle Sap (TS) had a much lower ^87^Sr/^86^Sr (0.708934) than M05, the closest Mekong mainstream site (0.710352), even if it belongs to the Phnom Penh Basin region [65]. This may be due to the different sources that affect the sites: Tonle Sap Lake is influenced by ^87^Sr/^86^Sr from the bedrock [65]. On the other hand, the Sekong River (SK) has a much lower ^87^Sr/^86^Sr (0.713943) than the nearby mainstream site (M04). The M04 site is influenced by ^87^Sr/^86^Sr ratio from the Mun River (Jurassic or Cretaceous Khorat plateau red-bed), while the Sekong River is influenced by a Permo-Triassic volcano-plutonic rock belt [65].

Although the ^87^Sr/^86^Sr in surface water reflects bedrock geology and remained constant across seasons and years [37, 67, 68], the ratios are possibly affected by groundwater [69] and rainfall [70–72], and by seawater, depending on the locations. The only slight variation in ^87^Sr/^86^Sr in saline waters limits the usefulness of this ratio to discriminate estuarine and marine waters [29]. In the Mekong Delta, the ^87^Sr/^86^Sr showed a logarithmic relationship with salinity from 5 to 30, but the variation was small (0.709174 to 0.709284) due to a short distance between the sites along the salinity gradient. However, the ^87^Sr/^86^Sr depended much more on the geology of the upstream origins than on salinity, making it possible to use this ratio to study fish movements in the fresh to estuarine waters along the Mekong River.

Among all the trace elements, the Ba/Ca ratio vs. Sr/Ca ratio was used to distinguish the different areas along the Mekong River because they both provide useable evidence of the origins of the water between the mainstream and tributaries or the different geological regions along the Mekong River [11]. The Ba/Ca ratio *vs*. Sr/Ca ratio in the different tributaries revealed differences that could be used to infer the origin of the water, and, consequently, the movements of fish between them [11, 73, 74]. The differences were particularly marked between the Mekong mainstream and its tributaries [11]. While Humston et al. [73] and Strohm et al. [74] showed that there was strong linear relationship between Sr/Ca and Ba/Ca in otoliths and water in various tributaries in the USA, the changes in otolith Sr/Ca and Ba/Ca did not reflect the seasonal river flow [37]. In the present study, the potential of comparing Sr/Ca and Ba/Ca between water and otoliths to determine the origin of fish origin remains questionable. The ^87^Sr/^86^Sr in the otoliths could be a better indicator and consequently more useful for discerning the natal origin and migration pathways of fish.

### Origin and movement of *Pangasius krempfi* along the Mekong River

The composition of trace elements in fish otoliths is influenced by different physiological processes that regulate their incorporation [18, 20, 22–24, 75, 76], and may thus limit its usefulness for inferring fish life history. However, some trace element ratios in the otolith, including the Sr/Ca or Sr isotope ratios, could reflect the ambient water conditions such as temperature and salinity [21, 47, 77] that are not strongly affected by biologically factors [28, 29] or seasonal-annual flows [78]. Therefore, otolith Sr/Ca and ^87^Sr/^86^Sr could be used as evidence to infer past environments the fish encountered during its lifetime.

Sr/Ca profiles of *Pangasius krempfi* otoliths from our previous study [47] had a very low ratio at the core, followed by a rapid increase, and high values during the rest of the fish’s life (Fig 8). The fact all individuals were born in freshwater environments was demonstrated, but without identifying the exact origin of the fish. The fact the fish remained in brackish water environments for the rest of their lifetime was also demonstrated. The aim of the present study was to more precisely identify the natal origin using the ^87^Sr/^86^Sr ratio along the Mekong River. Unlike the Sr/Ca, ^87^Sr/^86^Sr was the highest at the core, with more variance. These ^87^Sr/^86^Sr confirmed the natal origin in the freshwater environments of the Mekong River. The ^87^Sr/^86^Sr support the *P*. *krempfi* migration patterns between freshwater and brackish water in the Mekong Basin over their lifetime that we inferred from the Sr/Ca ratios in our previous study [14, 44, 45].

Combining otolith ^87^Sr/^86^Sr and Sr/Ca could provide the best evidence for the natal origin and migration behavior of anadromous species [79]. While otolith ^87^Sr/^86^Sr profiles showed the *P*. *krempfi* movements after hatching in freshwater regions, the otolith Sr/Ca profiles indicated possible movements and migrations between different saline environments. The present study demonstrated that *P*. *krempfi* is an anadromous fish, hatches in freshwater regions in the Mekong mainstream along the Laos–Thailand border or in Cambodia, and then migrates at early stages to the Mekong Delta, and spends most of its lifetime in brackish and marine environments. All *P*. *krempfi* caught in the Mekong Delta were born in the upper part of the Mekong mainstream, having migrated more than 1,500 km in the Mekong River mainstream, with cross-border movements at least between Laos-Thailand, Cambodia, and Vietnam.

Comparing the ^87^Sr/^86^Sr in the core with those in the water, indicated that *P*. *krempfi* are born in the Mekong mainstream and never in the tributaries. There could be spawning grounds along Laos–Thailand border, or in Cambodia, or even in the upper part of the river in Vietnam. Our results point to several spawning locations, but these were not precisely identified, because the ^87^Sr/^86^Sr in the otoliths corresponded to those in different locations in the upper part of the stretch along the Laos-Thailand border. The cluster analysis separated three contingents with distinct ^87^Sr/^86^Sr profiles in their otoliths. One contingent hatched around Kampong Cham (M05, Cambodia), Cao Lanh (M06, Vietnam), Cai Be (M07, Vietnam) or even in the upper part of distributaries (CC and HL), another contingent hatched in the upper region from Khone Fall (Cambodia) to Nong Khai (Thailand). The third contingent had a higher ^87^Sr/^86^Sr at the otolith core than that of any water collected in this study with the exception of water in the Sekong River, where the ratio was even higher. These fish could have hatched at upper Nong Khai (Thailand), near Vientiane the capital of Laos.

The main conclusion of the present study is that *P*. *krempfi* does not have only one natal origin. Although the spawning habitat requirements and biology of this species are not fully known, the previous hypothesis was that the species spawns in the Mekong mainstream near the rapids and pool systems around the Khone Falls [43, 44, 80, 81]. The spawning season is assumed to occur between June and August, when fishermen observed specimens with eggs in Laos, but has never been reported in other periods [81]. By quantifying the genetic diversity and structure of *P*. *krempfi* in the Mekong Delta, Duong and Nguyen [82] reported that there were different spawning groups of this species. We, therefore, predicted that Khone Falls is not this species only spawning ground and that the spawning grounds are probably rapids along the Mekong mainstream from Vietnam and Cambodia, to the Laos-Thailand border: spawning grounds could also exist in upper Cao Lanh (Vietnam), from Kandal province to Kampong Cham (Cambodia) [80], at Khone Fall (Laos), or along the Laos–Thailand border of the Mekong River. *P*. *krempfi* breeds in different locations along the Mekong River, but never in tributaries, brackish or saline environments.

After hatching in freshwater, all *P*. *krempfi* migrated to the Mekong Delta and settled in the estuarine area, but the timing of leaving the hatching areas differed between the contingents. Although some individuals moved directly to the estuary after hatching, this could be due to water currents flushing them before they had grown big enough to swim against the current [44]. Other individuals appeared to move around in their area of origin or freshwater regions, with slightly different ^87^Sr/^86^Sr, before migrating to the Delta. Chan et al. [80] reported two-month-old juveniles at Mukdahan (Thailand), consistent with the delayed migration recorded for some individuals in our study. Migration to the Mekong Delta probably depends on natural food sources, mainly composed of their favorite leaves and benthic food [44, 81], and subsequently on the abundance of riparian vegetation in the estuarine environments.

The purpose of the migration could be either moving from suitable reproduction habitats to more suitable feeding habitats for growth, or avoiding predators and adverse abiotic conditions upstream [83]. Poulsen et al. [14] proposed that the *P*. *krempfi* caught in the Mekong Delta belong to the upper parts of the Mekong system, and indicated possible migration routes. The marine residence and homing behavior of *P*. *krempfi* were observed by Baran et al. [5] and Hogan et al. [44], who reported that these fish are able to come return to their natal origin for spawning [44]. Two of the contingents settled in the estuarine habitats, but the fish in the other contingents moved frequently between estuarine water and slightly brackish water upstream (M08). These results agree with those of Hogan et al. [44] and Tran et al. [47], who reported several migratory clusters in a single population. During its life in estuarine waters, *P*. *krempfi* can move around different areas, but only occasionally to freshwater. The longest distance traveled between marine and freshwater environments after reaching the Mekong Delta was observed for two individuals who moved 40 km upstream near M08 (My Tho, Tien Giang Province). The other individuals only moved around the brackish and marine environments and did not reach the freshwater areas before capture.

In this study, spawning locations were predicted but not precisely identified, because the ^87^Sr/^86^Sr in the water overlapped in some locations, probably due to mixing between different sources. A larger-scale study of values, sources, and changes in water ^87^Sr/^86^Sr in the Mekong River could provide more data for applications of otolith chemistry in this area. The present study was also limited by studying only *P*. *krempfi* captured in the Mekong Delta and no evidence of homing behavior was found because no individual otolith showed a possible return to its freshwater origin during its lifetime. The *P*. *krempfi* analyzed in the present study were either non-homing individuals, or individuals who had not yet reached the reproductive stage, even if the maximum age was 5 years (total length 0.8 m, weight 4.75 kg). Although there have been several studies of the migration patterns of this species based on field observations, fish catches or otolith chemistry [5, 44, 45, 47, 84], the life cycle of *P*. *krempfi* in the Mekong River is still not fully understood. Age at first sexual maturity, age at migration, their behavior during and after spawning remain unknown. Other individuals captured upstream in Thailand and Laos, could provide more information on migrating pathways.

### Threats to and conservation of *Pangasius krempfi* and migratory fish in the Mekong River

Migratory fish are an important resource for many stakeholders along the Mekong River [45]. Understanding the early life history and the migratory behavior of these fish is crucial for identifying their nursery habitats and their migration pathways for the purpose of conservation and fishery management [85]. This study highlighted the ecological and anthropogenic threats to *P*. *krempfi*, as it is an obligate anadromous species. *P*. *krempfi* has been shown to be highly vulnerable to a combination of stresses including climate change, habitat loss, dam construction, pollution, and overexploitation [5, 12, 86, 87]. Rising sea levels, the combination of severe drought, saltwater intrusion in the coastal Mekong Delta in recent years and the construction of hydropower dams have reduced feeding habitats for the species at the adult stage [88–91]. Changes in rainfall patterns could also affect the water levels required for breeding and the water levels in migration pathways which, in turn, could affect breeding success [91–93]. Hydropower dams along the Mekong mainstream reduce the availability of rapids and pools, which are possible breeding habitats of this species. Future dams will increase the barriers along the migration pathways of the species, especially for breeding, and will isolate habitats of the required quality [92]. Ultimately, these threats will increase the mortality of adults and juveniles, compromising recruitment, and reducing population fitness [12, 13, 94]. These threats will not only impact anadromous fish, such as *P*. *krempfi*, but also diadromous and potamodromous fish and even non-migratory fish.

Dams in the Mekong basin have more impact on migratory fish species than upstream dams in China, and mainstream dams have a greater environmental impact than dams built on tributaries [12]. If hydropower dams are constructed on the mainstream Mekong in Cambodia and southern Laos (like the Sambor, Stung Treng, Don Sahong, Lat Sua and Ban Koum dams), they will be a major threat to fish communities in the Mekong River, especially anadromous species, such as *P*. *krempfi*, which migrate upstream to complete their life cycle. Dams further upstream and on tributaries will also have local impacts on short-range migratory fish [11, 12]. However, the effects of dam construction on *P*. *krempfi* populations need to be considered carefully as the wide range of breeding locations could reduce the impact of habitat disconnection on the populations. The lower *P*. *krempfi* contingent born around Phnom Penh and the Mekong Delta are hypothesized to not be directly affected in the mainstream because until now, no dam has been constructed between the delta and the sea. It is possible that the upper contingents may adapt to the dams by switching to lower spawning grounds to avoid migrating up or down from the dams.

Protection of hatching habitats, nursery grounds, and environmental connectivity along the migration pathways are key issues for fish conservation. Building fishways, which could mitigate the threats posed by dams and protect fish migration routes, ensuring habitat connectivity, and rehabilitating fisheries are possible solutions to cope with dam construction [94, 95]. However, the fish passages must be designed to suit the specific topographic and hydrological conditions of the river, as well as to respect the ecological and biological conditions required at the different life stages of the fish species [12, 95]. Artificial fish passages are therefore unlikely to be an effective mitigation measure in the Mekong River because of a large number of migration patterns, body sizes, shapes and behavior of migratory fish [96]. Finding a balance between environmental, economic, and social demands are key issues for the sustainable development of the Mekong region [12], and nature-based solutions are the best way to protect and conserve the area and the fish communities.

## Conclusion

This study demonstrated the use of ^87^Sr/^86^Sr ratios in water and fish otoliths to understand the life history cycle of an anadromous catfish caught in the Mekong River. ^87^Sr/^86^Sr has never previously been measured in the Mekong River for this purpose. The ^87^Sr/^86^Sr mapped in this study differed significantly between the Mekong mainstream and its main tributaries, and between different geological regions along the Mekong mainstream. The targeted species *Pangasius krempfi*, caught in the Mekong Delta, hatch in freshwater along the Mekong mainstream, from Phnom Penh (Cambodia) to Nong Khai (Thailand), or even further, before settling in the delta. Spawning habitats and migration pathways are threatened by habitat degradation and by the increasing number of hydropower dams along the river. Thus, the conservation of *P*. *krempfi*, as well as other migratory fish in the Mekong River, requires the agreement of and actions by all the stakeholder countries located along the Mekong River.

## Supporting information

S1 Fig Summary analysis process for trace element and ^87^Sr/^86^Sr in the water. (TIF)

S2 Fig Otolith of *Pangasius krempfi*. (A) Whole otolith of *Pangasius krempfi*, (B) the illustration for sliced transversally cutting (white bank) and (C) otolith slice with the laser transect from core to edge (dotted line).
(TIF)

S3 Fig A. Correlation between trace element concentrations and salinity in water collected along Mekong River and tributaries. B. Correlation between trace element concentrations and salinity in water collected along Mekong River and tributaries (continuous).
(TIF)

S1 Table Accuracy and precision of trace elemental analyses assessed using SRM 1640a reference material. LR represents low resolution, MR medium resolution and HR high resolution. RSD is calculated by the difference between measured and certified values divided by the certified value and expressed in percentage.
(DOCX)

S2 Table Trace element concentrations. Sampling locations, salinity and trace element concentrations measured by AETE-ISO OREME and IES.
(XLSX)

S3 Table ^87^Sr/^86^Sr values in otoliths of *Pangasius krempfi*. Distance from the core to the analyses points and ^87^Sr/^86^Sr values in all otoliths of *Pangasius krempfi*.
(XLSX)
