# Supplementary figures and images for: Natal origin and migration pathways of Mekong catfish (Pangasius krempfi) using strontium isotopes and trace element concentrations in environmental water and otoliths
Source: PLoS One. 2021 Jun 10;16(6):e0252769. doi: 10.1371/journal.pone.0252769 (PMC8191956; doi:10.1371/journal.pone.0252769)

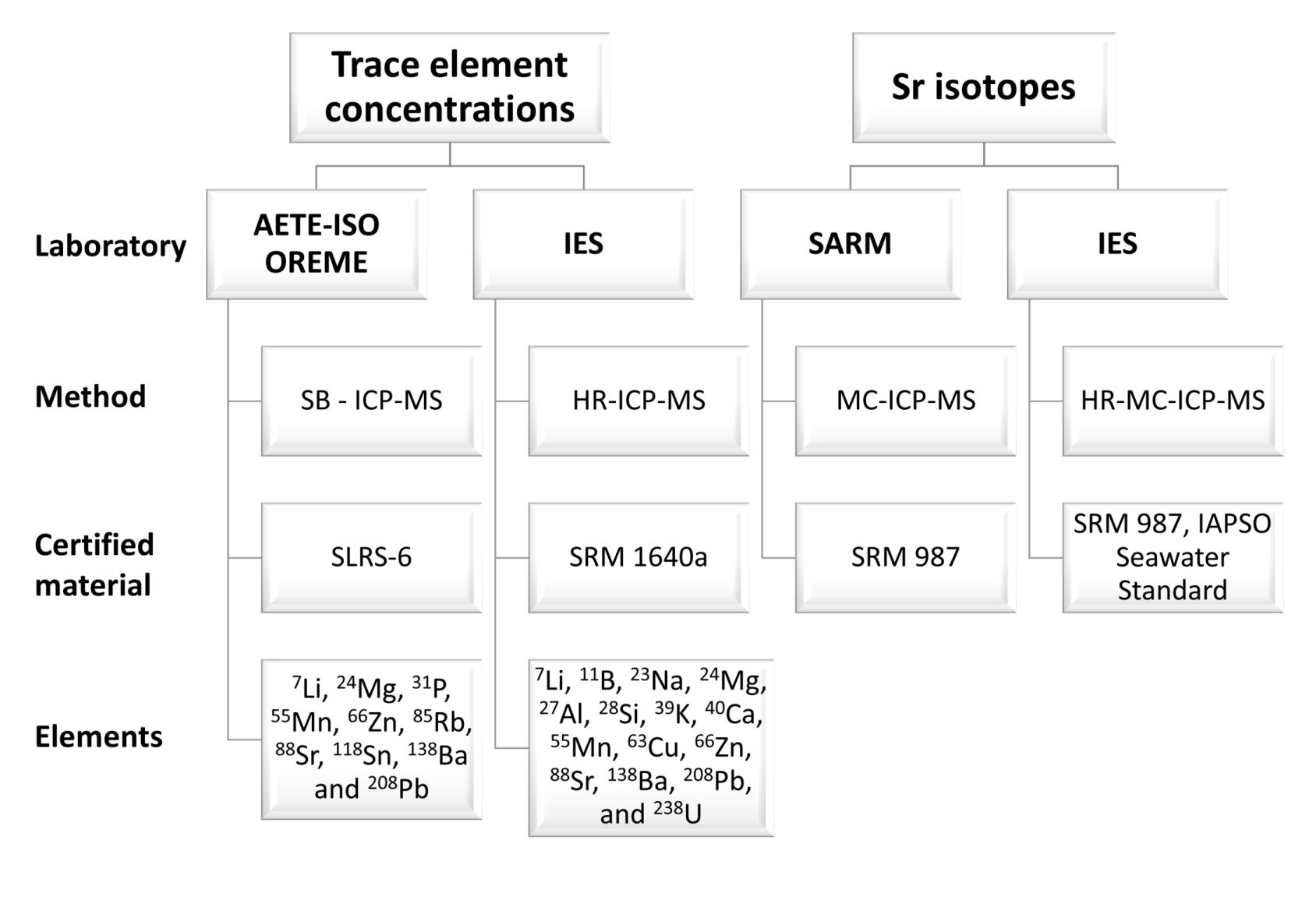

Supplement: S1 Fig — (TIF) [file pone.0252769.s001.tif]

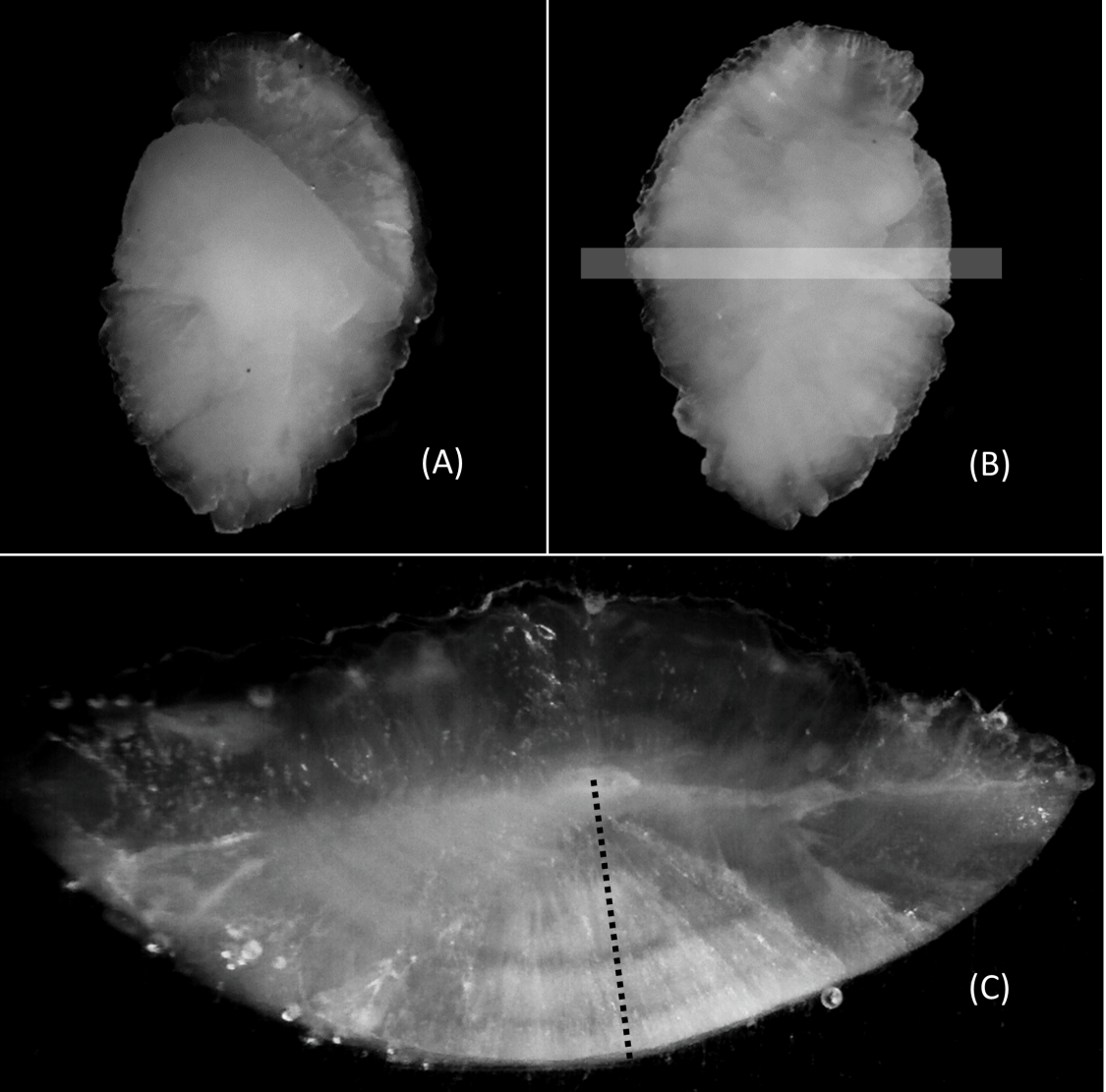

Supplement: S2 Fig — (A) Whole otolith of Pangasius krempfi, (B) the illustration for sliced transversally cutting (white bank) and (C) otolith slice with the laser transect from core to edge (dotted line). (TIF) [file pone.0252769.s002.tif]

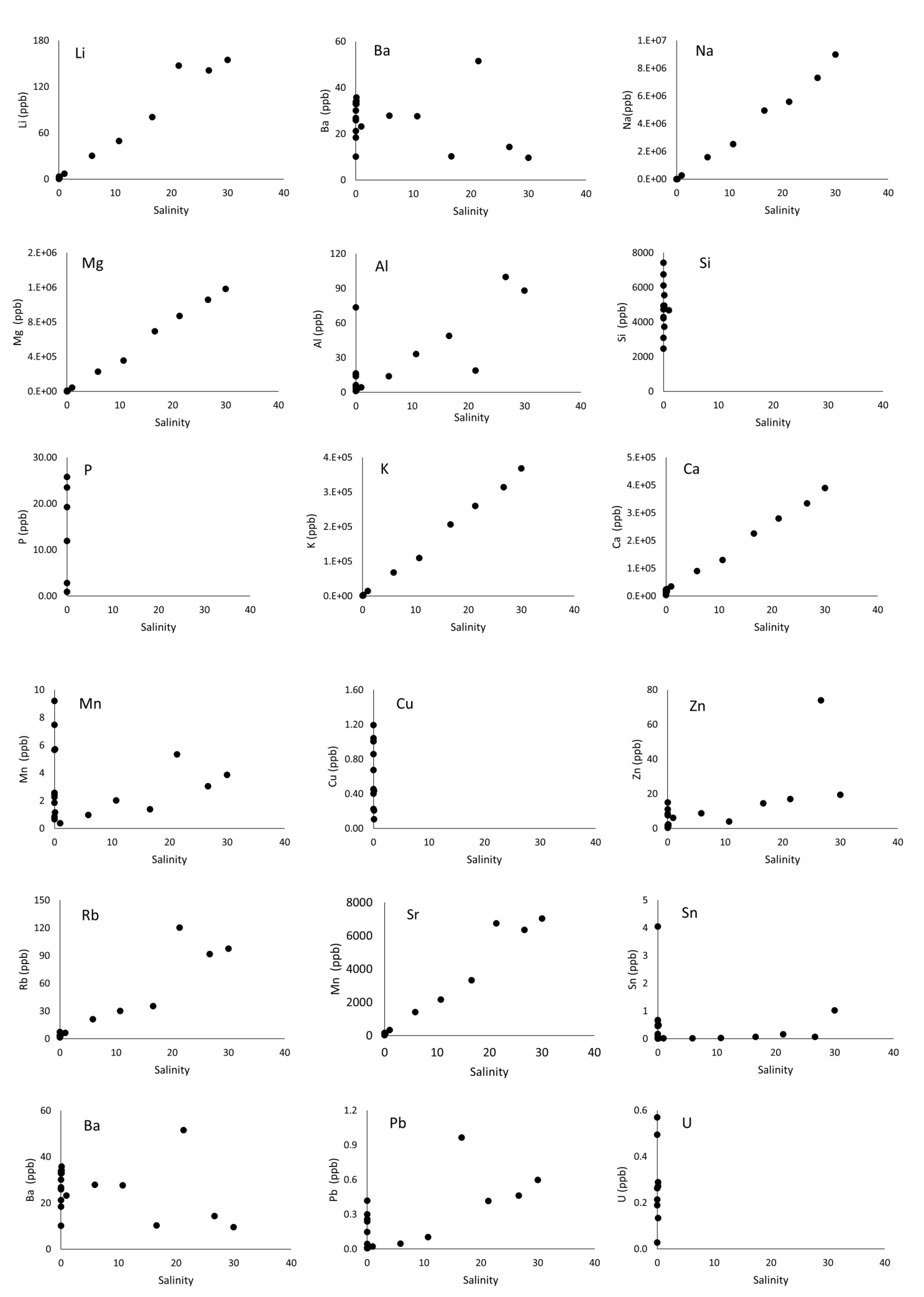

Supplement: S3 Fig — A. Correlation between trace element concentrations and salinity in water collected along Mekong River and tributaries. B. Correlation between trace element concentrations and salinity in water collected along Mekong River and tributaries (continuous). (TIF) [file pone.0252769.s003.tif]
