# Supplementary material for: Natal origin and migration pathways of Mekong catfish (Pangasius krempfi) using strontium isotopes and trace element concentrations in environmental water and otoliths
Source: PLoS One. 2021 Jun 10;16(6):e0252769. doi: 10.1371/journal.pone.0252769 (PMC8191956; doi:10.1371/journal.pone.0252769)
Supplement: S1 Table — LR represents low resolution, MR medium resolution and HR high resolution. RSD is calculated by the difference between measured and certified values divided by the certified value and expressed in percentage. (DOCX) [file pone.0252769.s004.docx]

|  | Li (ppb) | B (ppb) | Al  (ppb) | Cu  (ppb) | Zn  (ppb) | Sr (ppm) | Ba  (ppb) | Pb  (ppb) | U  (ppb) | Mg  (ppm) | Si  (ppm) | Ca  (ppm) | Mn  (ppb) | Na  (ppm) | K  (ppm) |
| --- | --- | --- | --- | --- | --- | --- | --- | --- | --- | --- | --- | --- | --- | --- | --- |
| Analysing mode | LR | LR | LR | LR | LR | LR | LR | LR | LR | MR | MR | MR | MR | HR | HR |
| Accuracy (RSD%) | 4.22 | 6.89 | 4.58 | 9.78 | 8.58 | 5.48 | 10.72 | 7.67 | 6.04 | 2.40 | 11.06 | 4.84 | 12.08 | 7.49 | 8.16 |
| Precision (1SD) | 0.085 | 0.348 | 0.142 | 0.076 | 0.120 | 0.001 | 0.110 | 0.013 | 0.007 | 0.032 | 0.016 | 0.0132 | -.115 | 0.043 | 0.008 |
